# Rapid Point-of-Care Diagnostic Test for Syphilis in High-Risk Populations, Manaus, Brazil

**DOI:** 10.3201/eid1504.081293

**Published:** 2009-04

**Authors:** Meritxell Sabidó, Adele S. Benzaken, Ệnio José de Andrade Rodrigues, Philippe Mayaud

**Affiliations:** Universitat Autònoma de Barcelona, Barcelona, Spain (M. Sabidó); Fundação Alfredo da Matta, Manaus, Brazil (A.S. Benzaken, E.J. de Andrade Rodrigues); London School of Hygiene and Tropical Medicine, London, UK (P. Mayaud)

**Keywords:** Syphilis, point-of-care diagnostic tests, high-risk populations, evaluation studies, prevention and control, Amazon, Brazil, dispatch

## Abstract

We assessed the acceptability and operational suitability of a rapid point-of-care syphilis test and identified barriers to testing among high-risk groups and healthcare professionals in a sexually transmitted infections clinic in Manaus, Brazil. Use of this test could considerably alleviate the impact of syphilis in hard-to-reach populations in the Amazon region of Brazil.

The new generation of rapid point-of-care (POC) syphilis diagnostic tests has shown good reliability and can be performed in any clinical setting. These tests can provide fast results during a patient’s initial visit (*1*).

Implementation of syphilis screening programs can be hampered by operational and technical difficulties (*2**–**4*) such as inadequate training, poor supervision, inconsistent quality control, disruptions in receiving medical supplies, and erratic electricity or refrigeration facilities needed to perform the test or store its reagents (*5*). Patients’ barriers to testing are often structural (accessibility and clinic hours) or financial (*4*). Further, test-seeking behavior can be negatively affected by the silent nature of the infection, the patient’s limited syphilis-related knowledge, and the perceived quality of healthcare provided. Overcoming any of these barriers would result in increased accessibility of services to those most in need and effective implementation of testing within often fragile healthcare systems located in resource-limited countries.

## The Study

**Figure 1:**
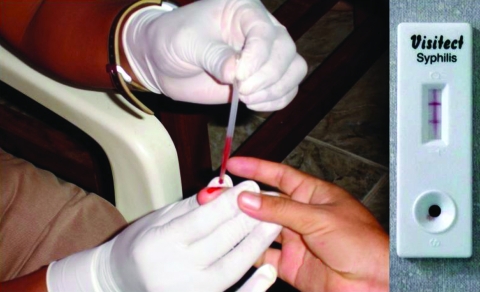
Rapid point-of-care syphilis test. Finger prick (left); diagnostic cassette with test bands results (right).

The study was undertaken within a larger field evaluation of a novel POC test for the detection of treponemal antibodies (VisiTect Syphilis, Omega Diagnostics, Alloa, Scotland) (Figure 1) in a sexually transmitted infections clinic located in a “red-light” area near the harbor of Manaus, Brazil (*6*). Before the evaluation, all staff were trained in the use of the test. One month after the start of syphilis screening, 10 clinical staff and 2 laboratory technicians were interviewed to identify factors that facilitated or impeded performance of the test.

Over a 6-week period, 60 patients, who had given informed oral consent, were interviewed while awaiting test results. The questionnaire was designed to examine reasons and potential barriers for syphilis testing, participant satisfaction with the information and attention received, and syphilis knowledge.

**Figure 2:**
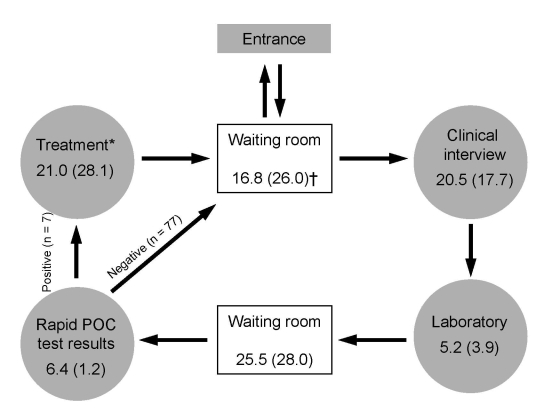
Time-flow analysis for point-of-care (POC) syphilis testing and treatment during a visit to a sexually transmitted infections clinic in a red-light area of Manaus, Brazil, 2006 (N = 84). Total time in minutes (SD) spent by patients completing all stages is shown, regardless of treatment. Average duration time spent at the health facility in mean (SD) minutes: 88.9 (37.1). *Only 7/84 (8.33%) of patients required to complete this stage; †includes time required to get into and to leave the health facility.

A separate subsample of consecutive patients, who were not interviewed, participated in a time-flow analysis. At all stages of the consultation, staff recorded, on forms given to the patient, the exact start and finish time of contact with the patient and the number of minutes required to perform each task with the patient (Figure 2). Time difference between tasks is the waiting time.

Descriptive analysis of quantitative data was done by using STATA version 9.0 (StataCorp, College Station, TX, USA). Detailed notes on qualitative items were analyzed thematically, coded, and categorized according to underlying themes included in the questionnaire. Categorized information was classified into 5 themes (confidence in test results, syphilis knowledge, test-seeking behavior, test preference, and evaluation of health services).

Most staff (10/12) thought training was satisfactory, and 9/12 reported test instructions as “perfectly easy” or “very easy” to follow. Laboratory technicians (2/2) found the test easy to use and interpret, requiring only ≈2 minutes to perform. In contrast, 2/10 physicians and nurses found interpretation of the test results “complex” or “not easy” because the test sometimes yielded a blurred result line difficult to assess and because the test could react and turn positive after the expected reading time (15 minutes). Most physicians and nurses (6/10) lacked confidence in the POC test result. They correctly pointed out that the POC test did not differentiate between past-treated and recent syphilis. Some staff reported finding discrepancies between the results of the POC test and conventional treponemal assays (*7*). Conventional treponemal assays, which rely on testing venous blood samples, were preferred by 6/10 clinical staff, were sometimes perceived to be less painful for the patients (4/10 clinicians responding), and provided more relevant information for patient care.

Sixty patients (36 women, 60%) were interviewed: 25 female sex workers (42%), mean age 31 years (SD, 10.5); 20 male clients of female sex workers (33%), mean age 44 years (SD, 15.0); and 15 (25%) other patients living or working in the Manaus harbor area, mean age 40.2 years (SD, 15.7). After an information campaign began (posters, street banners, flyers, and peer communication), patients took a median of 3.5 days (range 0–30) to attend the clinic; 20 (33%) participants sought testing on the same day they received the information.

Participants cited perceived risk for infection and knowledge of people who had already been tested as the primary motivators for testing. As one male harbor worker said, “I came for testing because some people said I was sick.” For three quarters of the participants (45/60), work schedules were not a limitation to seeking testing. Most respondents (69%) found that the time required for testing was short or very short. Almost half (48%) did not incur any costs in coming to the clinic for testing; others incurred only transportation costs. The rapid POC test did not cause any discomfort to 41 (68%) persons, but others found the fingerprick more painful and frightening than venipuncture. As one female sex worker remarked, “It (the fingerprick) is really itching!” Half (52%) of the respondents stated that they would choose the conventional test because this test was less painful, and they were accustomed to blood tests by venipuncture. Among patients who preferred rapid testing, the main reasons given were the rapidity of knowing their syphilis status and, for some, fear of needles. One female sex worker explained, “I am afraid of needles, and the fingerprick is better and much quicker!”

All respondents, with the exception of 2, trusted test results mainly because of their respect for the organization that ran the clinic. One male client commented, “I trust them because Fundação Alfredo da Matta is a serious organization that takes care of the human person and doesn’t care about the money.”

 Almost all participants classified the attention received as satisfactory and indicated the general caring attitude of staff and lack of stigmatization as remarkable qualities of the service (Table). Total mean time at the health facility in minutes was 88.9 (SD 37.1). Results of the time-flow analysis conducted among 84 patients showed that, excluding time spent receiving treatment for 7 (8.3%) patients, average time spent at the clinic was 51 minutes (SD 32) (Figure 2).

**Table:** Responses to selected questions on a questionnaire administered to 60 patients undergoing POC syphilis testing in an STI clinic, Manaus, Brazil, 2006*

Questions	No. (%) responses
Were you satisfied with services received?†	
5 out of 5 factors	56 (93)
4 out of 4 factors	4 (7)
Would you recommend the syphilis rapid test to friends?	
Yes	57 (95)
No	2 (5)
How would you rate the information received from clinical staff?	
Satisfactory	36 (60)
Difficult to understand	8 (13)
Did not receive information	16 (27)
How much do you know about syphilis?‡	
Could identify STI	12 (20)
Could explain some or all of its symptoms	12 (20)
Could explain some of its complications	5 (8)
Do you know how syphilis is transmitted?‡	
Unprotected sex	12 (20)
Sex regardless of condom use	19 (32)
Mother to child	6 (10)
Contaminated blood	13 (22)
Kissing	6 (10)
Sitting in the same place	3 (5)
Skin lesions	2 (3)
Do you know whether syphilis can be cured?‡	
Yes	53 (89)
No/don’t know	6 (11)

*POC, point of care; STI, sexually transmitted infection. †Measured on a scale from 0 (totally unsatisfactory) to 5 (totally satisfactory). ‡Open questions.

## Conclusions

From patient and laboratory technician perspectives, the rapid POC test was acceptable and operationally appropriate as a screening tool for diagnosis of syphilis and was performed within a reasonable waiting time for patients. However, of concern was the staff’s lack of trust in test results, which was correctly attributed to the test’s failure to differentiate between past-treated infections and active cases. Tests that could overcome this main technological handicap would be welcomed. Barriers to testing that need to be addressed are the pain caused by the finger puncture and poor knowledge of syphilis in a clearly high-risk population (*6*). The main study limitation is the possible selection bias of the target population who sought testing at the clinic.

The performance of the POC test combined with the advantage of on-site testing and same-day treatment are operational characteristics likely to improve coverage of syphilis screening in hard-to-reach populations such as highly stigmatized groups or those living in remote rural areas (*8*). Immediate, on-site testing is especially important for extending syphilis screening programs in the Amazon Region, a region characterized by long distances to most of its settlements, the need for river transportation, and the lack of well-equipped laboratories and trained technicians.
